# Hidden Contribution of Severe Gastroesophageal Reflux Disease to Recurrent Pneumonia in an Octogenarian With Prior Gastrectomy

**DOI:** 10.7759/cureus.91250

**Published:** 2025-08-29

**Authors:** Junnosuke Yoshiwara, Natsumi Yamamoto, Kurumi Kasai, Ryuichi Ohta

**Affiliations:** 1 Department of Community Care, Unnan City Hospital, Unnan, JPN

**Keywords:** 80 and over, aged, aspiration pneumonia, bacterial, dementia, gastrectomy, gastroesophageal reflux, pneumonia

## Abstract

We report the case of an 88-year-old man with a history of distal gastrectomy who developed recurrent episodes of pneumonia within short intervals despite appropriate antibiotic therapy. He presented with fever and increased sputum production only two days after discharge from a prior pneumonia admission. His medical history included Alzheimer’s disease, dilated cardiomyopathy, type 2 diabetes mellitus, and permanent atrial fibrillation. On admission, he was febrile and hypoxemic, with fine crackles on chest auscultation and new infiltrates on radiography and computed tomography. Despite the absence of overt swallowing dysfunction, the repeated recurrence prompted further evaluation. Upper gastrointestinal endoscopy revealed persistent Los Angeles grade C gastroesophageal reflux disease (GERD) despite proton pump inhibitor therapy. The patient also reported lying down immediately after meals, suggesting nocturnal and postprandial microaspiration as a key mechanism. Antibiotic therapy with ceftriaxone improved the pulmonary infiltrates, and he was discharged with reinforced reflux precautions and continued acid suppression therapy. Following discharge, he adhered to these lifestyle modifications and has not required readmission for pneumonia. This case highlights GERD as an underrecognized cause of recurrent pneumonia in elderly patients without obvious dysphagia. A comprehensive evaluation for GERD is crucial in managing unexplained recurrent pneumonia, particularly in patients with dementia, prior gastric surgery, or other aspiration risk factors.

## Introduction

Gastroesophageal reflux disease (GERD) is a chronic condition characterized by the reflux of gastric contents into the esophagus, most commonly presenting with symptoms such as heartburn and regurgitation [1]. The pathophysiology involves relaxation of the lower esophageal sphincter (LES), impaired esophageal motility, and delayed gastric emptying. In older adults, age-related declines in muscular strength, altered body posture, and comorbid conditions, such as obesity, diabetes, and neurological disorders, further increase the prevalence of GERD [2]. Moreover, anatomical changes following gastric surgery are known to exacerbate bile and pancreatic juice reflux, further elevating the risk of GERD development [3].

Persistent GERD may not only lead to esophageal complications but can also contribute to extra-esophageal manifestations, including chronic cough and recurrent respiratory symptoms [4]. Preventive strategies involving lifestyle modification and pharmacologic therapy are therefore essential to avoid disease progression and complications. However, in older patients, GERD often presents with atypical or subtle symptoms, which can delay diagnosis and appropriate treatment [5]. As a result, undiagnosed or poorly controlled GERD may predispose patients to recurrent aspiration and pneumonia, even in the absence of overt swallowing dysfunction.

Here, we report the case of an 88-year-old man with a history of distal gastrectomy who experienced recurrent pneumonia within a short period. Despite no apparent swallowing impairment on clinical examination, severe GERD was identified by upper endoscopy and confirmed as the primary cause of microaspiration leading to recurrent pneumonia, as supported by aspiration studies (e.g., videofluoroscopic swallowing study or scintigraphy). To our knowledge, GERD-induced pneumonia in an independent, very elderly patient without dysphagia is uncommon. This case highlights the importance of considering GERD as an underlying cause of recurrent pneumonia in older adults, particularly those with a history of gastric surgery.

## Case presentation

An 88-year-old man was referred to a rural hospital with fever. He had been discharged just two days earlier after treatment for pneumonia with ceftriaxone 2g/day for a week. On the day of presentation, he developed fever accompanied by increased sputum production and abnormal breath sounds, prompting his primary physician to suspect recurrent pneumonia and refer him to our facility. His medical history included Alzheimer’s disease, dilated cardiomyopathy, type 2 diabetes mellitus, and permanent atrial fibrillation. Notably, he had undergone a distal gastrectomy with Billroth-I reconstruction for a gastric ulcer approximately 40 years earlier. Over the past year, he had been admitted eight times for bacterial pneumonia. His long-term medications included edoxaban 15 mg, esomeprazole 20 mg, sennoside 12 mg, carvedilol 10 mg, and magnesium oxide 990 mg daily.

On arrival, he was alert and fully oriented. His vital signs were as follows: body temperature, 39.2 °C; blood pressure, 157/92 mmHg; heart rate, 100 beats/min; respiratory rate, 22 breaths/min; and peripheral oxygen saturation (SpO₂), 91% on room air. Chest auscultation revealed fine late-inspiratory crackles in the right lung field and inspiratory crackles throughout the left lung field. There was no evidence of jugular venous distension, weight gain, or peripheral edema suggestive of heart failure. Laboratory tests revealed mild anemia and elevated lactate dehydrogenase levels (Table 1).

**Table 1:** Initial laboratory data of the patient Abbreviations: CRP, C-reactive protein; FiO₂, fraction of inspired oxygen; PCO₂, partial pressure of carbon dioxide; PO₂, partial pressure of oxygen; HCO₃⁻, bicarbonate; BE, base excess; cLac, lactate concentration Laboratory reference ranges may vary slightly by institution. Urinalysis values reflect dipstick test results. Blood gas values were obtained via arterial blood sampling on room air (FiO₂ 21%).

Parameter	Level	Reference
White blood cells	8.3	3.5–9.1 × 10^3^/μL
Neutrophils	80.6	44.0–72.0%
Lymphocytes	12.5	18.0–59.0%
Red blood cells	3.0	3.5–9.1 × 10^6^/μL
Hemoglobin	9.5	11.3–15.2 g/dL
Hematocrit	28.6	33.4–44.9%
Mean corpuscular volume	93.5	79.0–100.0 fl
Platelets	21.4	13.0–36.9 × 10^4^/μL
Total protein	7.1	6.5–8.3 g/dL
Albumin	3.0	3.8–5.3 g/dL
Total bilirubin	0.4	0.2–1.2 mg/dL
Aspartate aminotransferase	34	8–38 IU/L
Alanine aminotransferase	14	4–43 IU/L
Lactate dehydrogenase	226	121–245 U/L
Blood urea nitrogen	13.6	8–20 mg/dL
Creatinine	0.84	0.40–1.10 mg/dL
Serum Na	143	135–150 mEq/L
Serum K	3.7	3.5–5.3 mEq/L
Serum Cl	112	98–110 mEq/L
CRP	12.3	<0.30 mg/dL
Urine test	-	-
Leukocyte	3+	Negative
Protein	Negative	Negative
Blood	Negative	Negative
Blood gas analysis	-	-
FiO2	21	%
pH	7.49	7.35-7.45
PCO2	29.6	35.0-45.0mmHg
PO2	63.3	75.0-100.0mmHg
HCO3-	22.8	20.0-26.0 mmol/L
BE	0.0	-3.0-3.0 mmol/L
cLac	1.3	0.5-1.6 mmol/L

**Figure 1:**
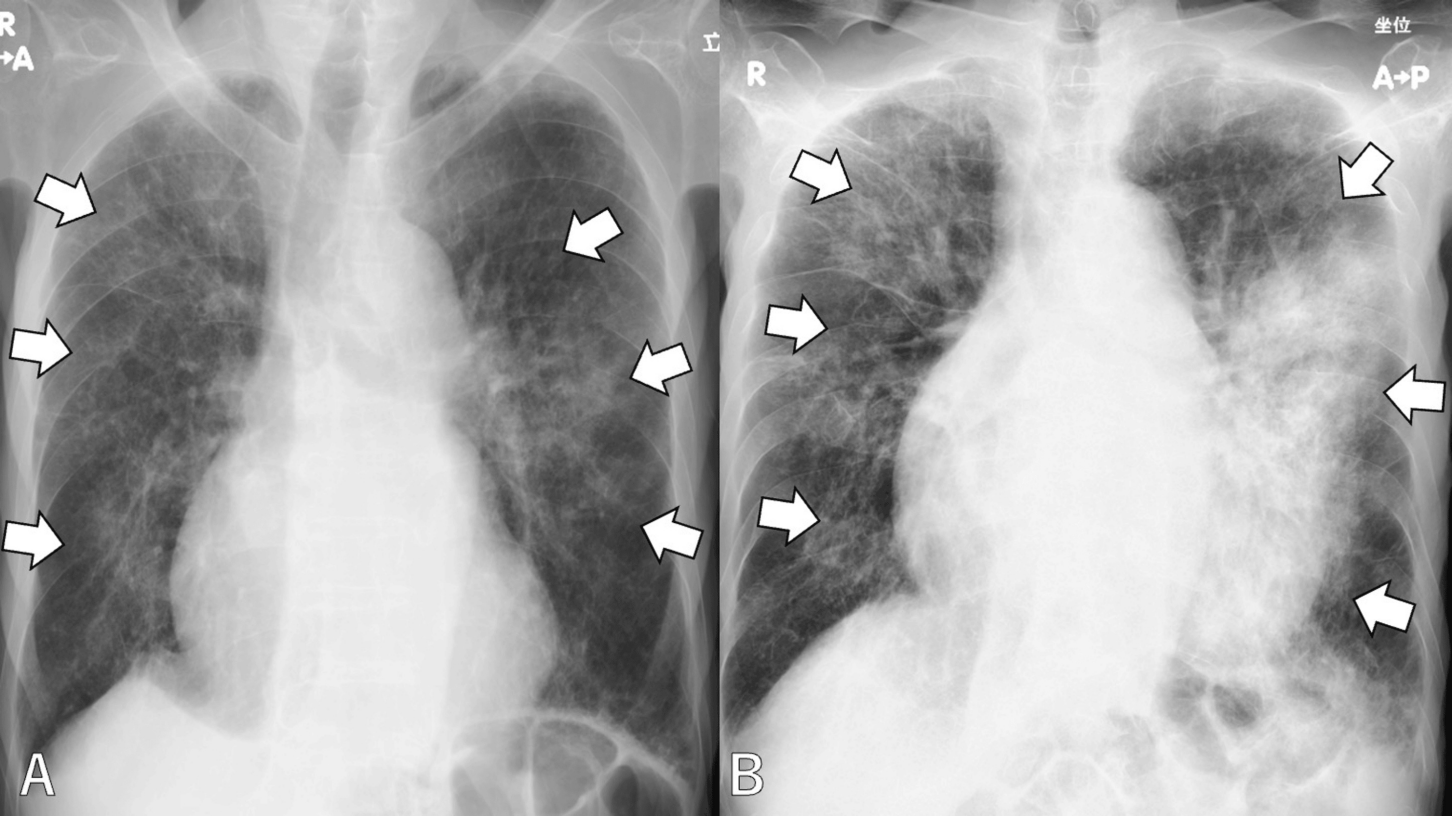
Chest radiography showing worsening consolidation in the left mid-lung field (B) compared with the previous imaging at discharge (A), as well as a new ground-glass opacity in the right middle-to-lower lung zones (white arrows)

Chest radiography showed worsening consolidation in the left mid-lung field compared with the previous imaging at discharge, as well as a new ground-glass opacity in the right middle-to-lower lung zones (Figure 1).

**Figure 2:**
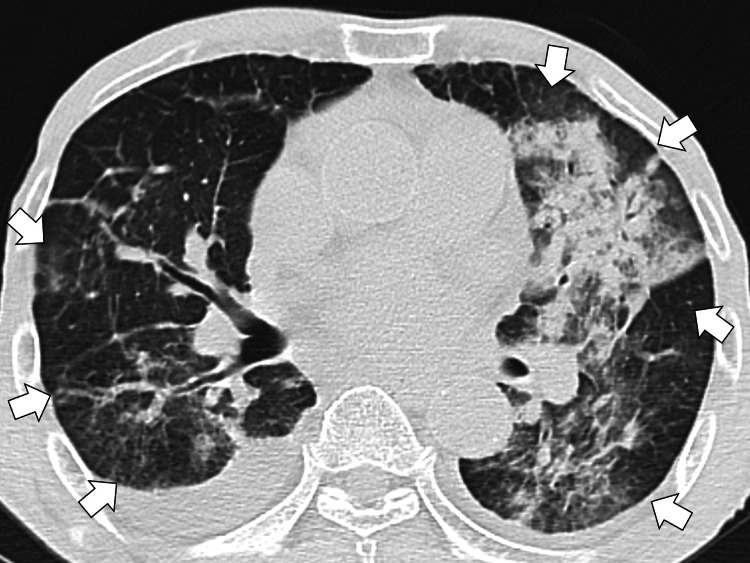
Chest computed tomography further confirming sputum impaction and infiltration on both lungs (white arrows)

Chest computed tomography (CT) further confirmed sputum impaction and infiltration in both lungs (Figure 2).

**Figure 3:**
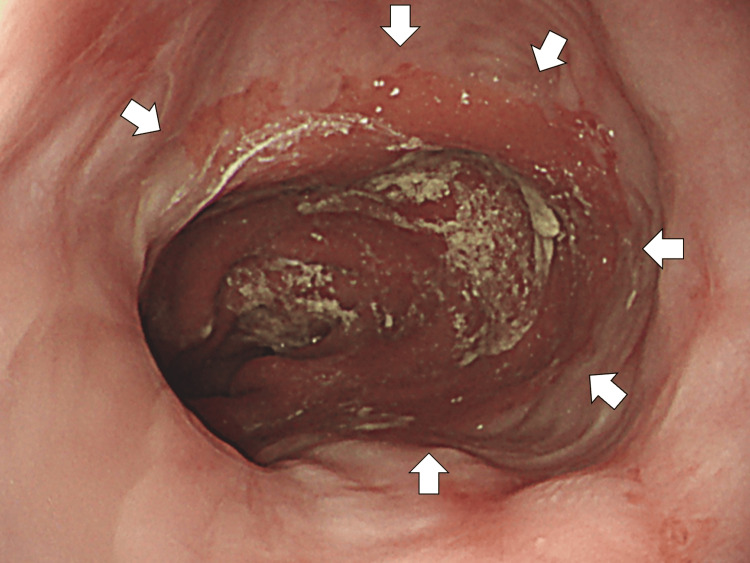
Endoscopy revealing persistent severe gastroesophageal reflux disease, classified as Los Angeles grade C, despite ongoing proton pump inhibitor therapy (white arrows)

The patient was diagnosed with recurrent bacterial pneumonia. Empirical antibiotic therapy with intravenous ceftriaxone of 2 g/day was initiated on hospital day 1. On day 3 of admission, a videofluoroscopic swallowing examination (VE) was performed, confirming no dysfunction of swallowing. Additionally, bedside aspiration tests and assessment by the Speech and Language Therapy (SLT) team revealed no abnormalities. Given the unusually short interval between the previous pneumonia episode and the current relapse, an upper gastrointestinal endoscopy was performed on hospital day 5 for further evaluation. The endoscopy revealed persistent severe GERD, classified as Los Angeles grade C, despite ongoing proton pump inhibitor therapy (Figure 3).

Notably, the patient reported that he frequently lay down immediately after meals. Taken together, microaspiration secondary to GERD was strongly suspected as the underlying cause of recurrent pneumonia.

**Figure 4:**
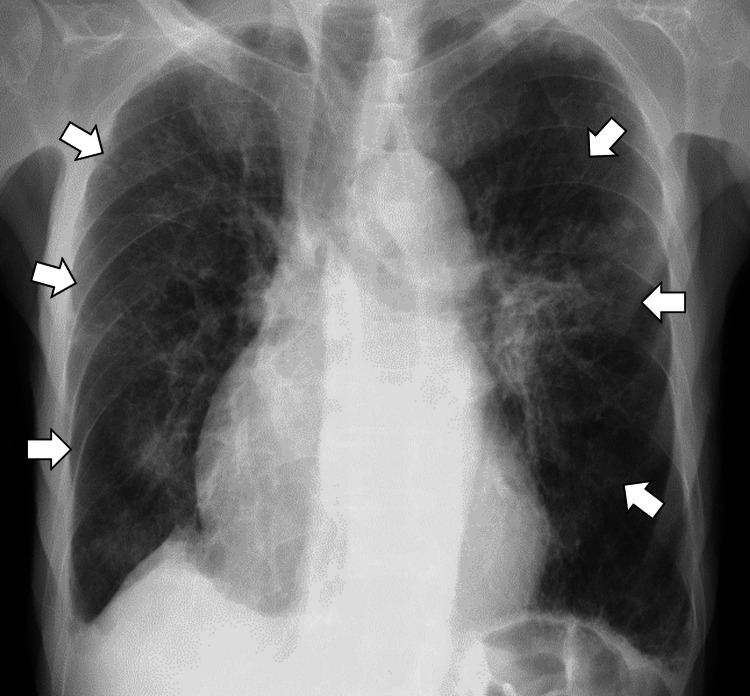
Follow-up chest radiography demonstrating significant resolution of the pulmonary infiltrates by hospital day 7

Follow-up chest radiography demonstrated significant resolution of the pulmonary infiltrates by hospital day 7, at which point ceftriaxone therapy was discontinued (Figure 4).

The patient was discharged home in stable condition on hospital day 10 with reinforced instructions regarding postprandial posture modification and continued acid suppression therapy. To prevent reflux, he was instructed to remain seated for at least two hours after meals, which he adhered to consistently. Since then, he has not required any further hospitalizations for pneumonia.

## Discussion

Ryu et al. reported that gastroesophageal reflux disease (GERD) was present in 32 of 52 patients (62%) with biopsy-proven aspiration pneumonia, suggesting that GERD frequently coexists with aspiration-related pulmonary infections [6]. Similarly, Noguchi et al. demonstrated that in elderly patients with aspiration pneumonia, the cumulative number of aspiration risk factors, including impaired consciousness, prolonged bed rest, cerebrovascular disease, dementia, sedative use, and GERD, was strongly associated with recurrence and mortality [7]. In the present case, the patient had two key aspiration risk factors, dementia and GERD. According to Noguchi’s cohort, patients with two risk factors have a 33% recurrence rate of pneumonia, a 5.4% 30-day mortality rate, and a 24.5% 6-month mortality rate [7]. These findings underscore the importance of proactive GERD management in aspiration-prone patients to reduce morbidity and mortality.

Beyond pneumonia, GERD has also been implicated in the pathogenesis of respiratory conditions such as bronchial asthma and chronic obstructive pulmonary disease [8]. In this patient, dementia and severe GERD likely acted synergistically, leading to recurrent pneumonia despite the absence of overt swallowing dysfunction. This case highlights that, in elderly patients with recurrent pneumonia, comprehensive aspiration risk assessment should include not only formal swallowing evaluation but also active screening for GERD as a potentially modifiable factor.

Although GERD has been identified as an independent risk factor for pneumonia, with an adjusted hazard ratio of 1.48 (95% confidence interval: 1.31-1.67) [9], case reports in which GERD is identified as the direct cause of pneumonia remain scarce. This is likely because reflux-associated aspiration is often silent, making it challenging to demonstrate a direct causal link in routine clinical practice. Swallowing function tests, such as videofluoroscopic or endoscopic evaluations, are typically used to detect overt aspiration. However, nocturnal microaspiration, which is highly relevant in aspiration pneumonia, may require prolonged esophageal pH monitoring for detection [10].

In most clinical scenarios, pneumonia improves promptly with appropriate antibiotic therapy, leaving little impetus for further investigation into underlying reflux [11]. In the present case, the unusually short interval between successive pneumonia episodes prompted further evaluation beyond conventional aspiration and airway infection mechanisms. Endoscopy revealed persistent severe GERD despite proton pump inhibitor therapy, and lifestyle factors, such as lying supine immediately after meals, were identified as additional contributors. This comprehensive assessment allowed GERD to be recognized as the primary underlying cause of recurrent pneumonia.

This case emphasizes that GERD should remain a differential consideration in elderly patients with unexplained recurrent pneumonia, particularly in those with a history of gastric surgery, dementia, or other aspiration risk factors [12]. Recognizing GERD as a hidden contributor may enable targeted interventions that prevent further respiratory complications [13]. In this patient, adherence to postprandial sitting and reflux precautions led to the successful prevention of pneumonia recurrence, underscoring the importance of simple but effective lifestyle modifications alongside pharmacological treatment for the prevention of readmission [14].

General physicians should be vigilant for occult aspiration pneumonia in older patients without new symptoms. In rural contexts, as societies age, an increasing number of older patients visit rural general hospitals, presenting with various vague symptoms that cause diagnostic difficulties [15,16]. Rural general physicians should promote their care through collaboration with multiple medical professionals [17].

## Conclusions

We report a rare case in which GERD, secondary to distal gastrectomy, was identified as the principal cause of recurrent pneumonia via microaspiration in a super-elderly patient. This case highlights the importance of evaluating not only swallowing dysfunction but also GERD in elderly patients with repeated episodes of pneumonia. Comprehensive GERD management, including lifestyle modification and optimized pharmacologic therapy, may reduce pneumonia recurrence. Particular attention should be given to patients with a history of upper gastrointestinal surgery, as they are at increased risk of severe reflux and its respiratory complications.
